# Reassessing the role of wild birds in the spread of antibiotic resistance: the white stork as a model species in studying populations from Central European river valley

**DOI:** 10.1128/spectrum.00990-25

**Published:** 2025-10-01

**Authors:** Andżelina Łopińska, Alicja Nowak-Zaleska, Jakub Z. Kosicki, Leszek Jerzak, Alicja Węgrzyn, Grzegorz Węgrzyn

**Affiliations:** 1 Institute of Biological Sciences, University of Zielona Góra 703861 , Zielona Góra, Poland; 2 Department of Biological Foundations of Physical Culture, Kazimierz Wielki University 56783, Bydgoszcz, Poland; 3 Department of Avian Biology and Ecology, Adam Mickiewicz University 49562 , Poznań, Poland; 4 Phage Therapy Center, University Center for Applied and Interdisciplinary Research, University of Gdańsk 49646, Gdańsk, Poland; 5 Department of Molecular Biology, Faculty of Biology, University of Gdańsk 49646, Gdańsk, Poland; Panepistemio Thessalias Tmema Geoponias Ichthyologias kai Ydatinou Periballontos, Volos, Greece

**Keywords:** animal vectors, antibiotic resistance, migratory birds, pathogenic bacteria, white stork, wildlife environment

## Abstract

It is commonly accepted that the emergence of multidrug-resistant bacteria in the hospital environment, in food production, and in the wild is the consequence of the overuse of antibiotics. The presence of antibiotic-resistant bacterial strains was reported in both wild and farmed animals. It was suggested previously that wild birds may be carriers of antibiotic-resistant bacteria acquired from agriculture and/or industrial/urban habitats. Here, we used the white stork (*Ciconia ciconia*) as a model species to assess the presence and origin of antibiotic-resistant bacteria in a breeding population located in river valley landscapes of western Poland. Cloacal swabs from 50 nestlings across 19 nests were collected during the 2019 breeding season, along with 49 environmental samples (soil, water, and food items) from known foraging sites. A total of 147 bacterial isolates representing 49 species were obtained. The majority originated from cloacal samples (58.5%), followed by water (16.3%), food (15.0%), and soil (10.2%). Resistance testing against 19 antibiotics revealed significantly higher resistance in food-derived isolates, while no significant differences were found among soil, water, and cloacal isolates. Correlation analyses based on resistance profiles showed no significant relationship between cloacal isolates and those from soil (rs = 0.11; *P* = 0.647) or food (rs = 0.25; *P* = 0.289), but a strong correlation with water isolates (rs = 0.68; *P* = 0.001). These results might suggest that wild birds’ role in transmitting antibiotic-resistant microorganisms might be more limited than predicted previously. Therefore, the role of wild birds, especially the white stork, in the transmission of antibiotic-resistant bacteria appears unclear and requires further consideration and re-examination.

**IMPORTANCE:**

Due to the overuse of drugs during animal breeding and the use of manures from breeding farms, antibiotic residues are present in the natural environment, especially in soil and water reservoirs. This causes a selection pressure on bacteria, creating a reservoir of antibiotic-resistant bacteria. It was suggested previously that wild birds may be carriers of antibiotic-resistant bacteria acquired from agriculture and/or industrial/urban habitats. Here, using a model of the white stork as a synanthropic bird, we asked whether this species is colonized by antibiotic-resistant bacteria, and if there is any significant correlation between bacteria present in samples of cloacal swabs and environmental samples from their habitats. We found no significant correlations between antibiotic-resistance patterns in bacteria isolated from white stork cloaca and those identified in bacteria isolated from soil and stork’s food. Thus, roles of wild birds in transmitting antibiotic-resistant microorganisms might be constrained and less significant than predicted previously.

## INTRODUCTION

The emergence of multidrug-resistant bacteria in the wildlife environment has been widely described and studied in the literature (1). It was concluded that the presence of multidrug-resistant strains in the environment is a result of the achievements of medicine, veterinary medicine, mass animal breeding and the food industry, and resultant overuse of antibiotics (2–4). Especially the massive development of agriculture and breeding animals for consumption has become major sources of antibiotic residues in the environment (5–9). Due to the widespread use of drugs during animal breeding and then the use of manures from breeding farms as agricultural fertilizer, antibiotic residues are present in the natural environment, especially in water (also in wastewater) and soil reservoirs (10). Antibiotic residues were also detected in animal-derived food (11). Their presence in the natural environment puts selection pressure on bacteria, creating a reservoir of environmental antibiotic-resistant bacteria (12).

The presence of subinhibitory concentrations of antimicrobials in the environment and the occurrence of antibiotic-resistance genes in bacteria occurring in the natural environment can create the opportunity for the transfer of these genes between resistant bacteria (donors) and the susceptible (recipient) ones (13). In recent years, an increasing number of reports on the occurrence of antibiotic-resistant bacteria in water, soil, and other habitats have been published. For example, in the Danube River, *Acinetobacter* spp. isolates were found to be resistant to carbapenems (meropenem and imipenem), piperacillin/tazobactam, and ceftazidime (14). Genes responsible for resistance to β-lactams, MLS antibiotics, sulfonamides, and tetracyclines were detected in samples from the Pilica River in Poland (15). Resistance genes specific to tetracycline, streptomycin, and erythromycin were found in agriculture soil samples in Poland (16). Moreover, chemical analyses revealed the presence of metoclopramide, sulfanilamide, salicylic acid, metoprolol, sulfamethazine, nimesulide, carbamazepine, trimethoprim, propranolol, and paracetamol in soil samples near poultry farms in Poland (17).

However, the problem is global as tetracycline-, aminoglycoside-, chloramphenicol-, and vancomycin-resistance genes were found in various habitats in many different parts of the world (18). The migration of resistant strains and genes leads to their appearance in various parts of the world, even in areas potentially free from anthropogenic influence (19–21). Many studies point to migratory wild bird populations as vectors for the spread of resistant strains, allowing such strains to persist in the environment for some time (22, 23).

Initially, the presence of multidrug-resistant strains of bacteria in wild or domesticated animals was reported. At the same time, there was an alarming risk that these bacteria could be transmitted to humans and, as a consequence, could spread infections in their environment (24–26). Wild birds can use a wide range of habitats because of their adaptation to flying. Due to their long-distance migratory routes, birds were suggested to be possible vectors of the global spreading of antibiotic-resistant bacteria (27–31). Such a proposal was based on various environmental observations. For example, carbapenemase-producing *Escherichia coli* (CPE) was first detected in Alaska from isolates obtained from gull feces (*Larus hyperboreus, Larus glaucescens, and Larus argentatus*). From almost 1,000 isolates, CPE prevalence was 0.5%, 2.2%, and 2.8% at the Soldotna landfill, lower Kasilof River, and Anchorage mudflats, respectively (32). Different rates of resistance of enterococcal species for tetracycline, erythromycin, ciprofloxacin, kanamycin, and chloramphenicol were detected in isolates from common buzzards (*Buteo buteo*) in Spain. *Escherichia coli* isolates showed high levels of resistance to streptomycin and tetracycline, whereas enterococci isolates represented high levels of resistance to tetracycline and erythromycin (33). The presence of extended-spectrum β-lactamase (ESBL)-producing *Enterobacteriaceae*, including the CTX-M-1 group, was detected among isolates from yellow-legged gulls (*Larus michahellis*) in France. As many as 9.4% of these isolates were classified as ESBL-type β-lactam resistant. Nine *E. coli* and one *Enterobacter cloacae* isolate belonged to the CTX M-1 group (34). Results of studies conducted in Portugal on multidrug-resistant bacteria demonstrated that wild birds can acquire bacteria resistant to antimicrobial compounds from the environment where humans were present. Thus, it was suggested that human activities are a source of multidrug-resistant bacteria in that area. Gulls, as long-distance migratory birds, might transfer such bacteria to other places because of using the same location (35). Such a phenomenon was also reported in studies on other bird species, like Russian rooks (*Corvus frugilegus*) in the Czech Republic (36), birds of prey in Germany and Mongolia (37), bar-headed geese (*Anser indicus*), and great black-headed gulls (*Larus marinus*) in China (38).

White stork (*Ciconia ciconia*) might be a valuable model species for research on wild birds as potential vectors in spreading antibiotic-resistant bacteria from agriculture and industrial/urban environments. This species has co-existed with humans for ages, making it typically synanthropic, long-distance migratory bird species (39). Moreover, white storks use arable lands, pastures, and banks of rivers as foraging areas. Storks use agricultural fields depending on the time of implementation of agrotechnical treatments. The use of pastures as foraging areas by white storks relies on the time of cattle grazing or grass cutting (40). Natural manures from large-area farms are contaminated by antibiotic residues. Places like this are a potential source and space of antibiotic-resistance gene transfer between bacteria in white stork and the environment (in both directions). It was reported previously that in isolates from storks, one can detect antibiotic-resistant strains, like *E. coli* strains resistant to β-lactams, *Campylobacter* spp. strains with ciprofloxacin- and tetracycline-resistance genes, and an opportunistic pathogen *Acinetobacter baumannii* (41, 42). Studies conducted in Spain indicated that storks foraging in landfill areas and within cities can be colonized by resistant strains. Notably, the bacterial species composition in these birds differed from that found in gulls, suggesting species-specific differences in the acquisition and spread of antibiotic-resistant bacteria. Among the potentially pathogenic strains identified, there were *Helicobacter* spp., *Enterococcus* spp., *Pseudomonas* spp., and various members of the *Enterobacteriaceae* family (41). Additional research from the same region has demonstrated that storks foraging in or near landfills are frequently exposed to *E. coli* strains resistant to cephalosporins. Among these isolates, a wide array of resistance genes was identified, including *bla*_CTX−M−1_, *bla*_CMY−2_, *bla*_CTX−M−14_, *bla*_SHV−12_, *bla*_CTX−M−15_, *bla*_CTX−M−32_, *bla*_CTX−M−1_, *bla*_CMY−2_, and *bla*_CTX−M−1_ (43). Similar findings were reported for ESBL strains (44). In a separate study, up to 50% of stork-derived *E. coli* isolates exhibited a multidrug-resistant (MDR) profile, with resistance observed against penicillin, aztreonam, quinolones, tetracycline, and gentamicin. The highest prevalence of antimicrobial resistance (AMR) was found for β-lactams, quinolones, and tetracyclines—drug classes widely used in both human and veterinary medicine in Spain (45). Furthermore, a recent study suggested that both storks and gulls may increase their carriage of antibiotic-resistant bacteria when shifting foraging behavior from paddy fields to landfill sites (46).

Nevertheless, on the basis of a recent comprehensive analysis of the available data in the literature, it was suggested that fears of pathogen spillover from wild birds to humans might be overestimated, especially when considering pathogenic bacteria (30). Therefore, it is still unclear whether the presence of antibiotic-resistant bacteria in stork-derived biological material represents a natural phenomenon, not affected by human civilization, or it reflects agriculture- and/or industry-related changes in the composition of microbiota in soil and/or waters with over-representation of antibiotic-resistant microorganisms. If the latter is true, the composition of antibiotic-resistant bacteria in the stork-derived material should be similar to that in the soil and water from the tested environment, indicating that wild birds are essential vectors in transmitting bacterial antibiotic resistance. In contrast, a lack of significant similarity in the composition of antibiotic-resistant bacteria between stork- and agriculture/industry-derived sources would suggest that these birds are less important in spreading antimicrobial resistance than previously assumed. Therefore, in this work, we aimed to test whether white storks, living in a natural region situated near urban and agricultural areas, are colonized by antibiotic-resistant strains of bacteria and if there is any significant relationship between such bacteria (present in samples derived from white storks) and environmental samples from their foraging area.

## RESULTS

### General distribution of bacteria

**Fig 1:**
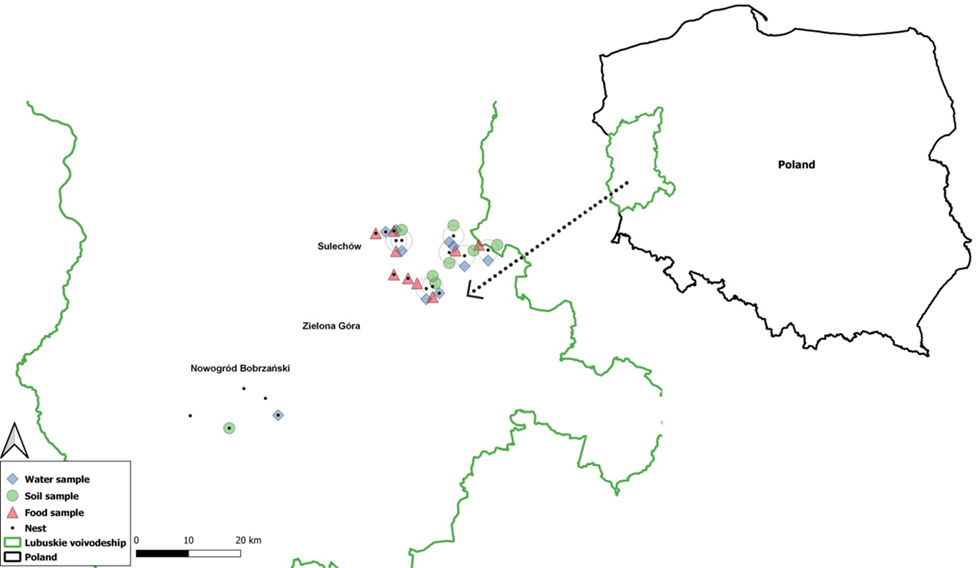
Location of white stork nests and environmental sampling sites. The green line shows the border of the Lubuskie Voivodeship (western Poland). The nest sites are marked with black dots. The blue squares, green circles, and red triangles indicate where water, soil, and food samples were collected. The environmental samples were collected in the area where storks foraged, within a radius of 1.5 km from the nest.

To test whether wild birds may serve as significant carriers of antibiotic-resistant bacteria acquired from agricultural and/or urbanized environments, we selected the white stork as a model species. The study was conducted in rural areas surrounding a medium-sized city, characterized by a mosaic of farmland, pastures, and natural habitats (Fig. 1). Such conditions provided suitable foraging and breeding grounds for white storks. Details of the study area are provided in *Materials and Methods*.

During the study, we isolated microorganisms belonging to 49 bacterial species from 11 families. They were isolated from cloacal swabs of white storks and from soil, water, and food sources that these birds potentially use during the breeding season (Table 1). In total, 147 isolates were collected; among them, 86 were from cloacal swabs, 24 from water, 22 from food, and 15 from soil (Table 1). Therefore, one can calculate that the cloacal material included the highest proportion of isolates (58.5%), whereas soil samples provided the lowest proportion (10.2%). Water and food samples accounted for about 16.3% and 15.0% of the isolates, respectively. Within these communities, *Enterobacteriaceae* predominated, with *E. coli* constituting 23.8% of all isolates; other frequently identified genera included *Pseudomonas*, *Proteus*, *Aeromonas*, and *Enterococcus* (Table 1).

**TABLE 1:** Bacterial isolates and their prevalence in white stork cloaca (*n* = 50), food (*n* = 16), water (*n* = 19), and soil (*n* = 19) of the foraging area (the order is according to prevalence)

Family prevalence (%)	Bacterial species	Number of isolates	Prevalence (%)^*a*^			
			Cloacal swabs	Food	Water	Soil
*Enterobacteriaceae* (44.90)	*Escherichia coli*	37	23.81	–	1.36	–
	*Escherichia hermannii*	3	2.04	–	–	–
	*Escherichia vulneris*	1	0.68	–	–	–
	*Enterobacter cloacae*	6	4.08	–	–	–
	*Citrobacter freundii*	6	4.08	–	–	–
	*Citrobacter amalonaticus*	1	0.68	–	–	–
	*Citrobacter braakii*	1	0.68	–	–	–
	*Klebsiella oxytoca*	2	–	–	1.36	–
	*Klebsiella pneumoniae*	2	–	–	1.36	–
	*Klebsiella* sp.	1	–	0.68	–	–
	*Raoultella ornithinolytica*	2	0.68	–	–	0.68
	*Raoultella planticola*	1	–	–	0.68	–
	*Salmonella enterica*	2	1.36	–	–	–
	*Leclercia adecarboxylata*	1	0.68	–	–	–
*Pseudomonadaceae* (19.73)	*Pseudomonas koreensis*	6	–	3.40	–	0.68
	*Pseudomonas aeruginosa*	4	2.72	–	–	–
	*Pseudomonas flavescens*	4	2.04	–	–	0.68
	*Pseudomonas jessenii*	3	–	2.04	–	–
	*Pseudomonas fulva*	2	1.36	–	–	–
	*Pseudomonas corrugata*	2	–	1.36	–	–
	*Pseudomonas thivervalensis*	2	–	1.36	–	–
	*Pseudomonas* sp.	1	0.68	–	–	–
	*Pseudomonas antarctica*	1	–	0.68	–	–
	*Pseudomonas extremorientalis*	1	–	0.68	–	–
	*Pseudomonas frederiksbergensis*	1	–	0.68	–	–
	*Pseudomonas putida*	1	–	–	0.68	–
	*Pseudomonas synxantha*	1	–	0.68	–	–
*Morganellaceae* (11.56)	*Proteus mirabilis*	14	7.48	–	–	2.04
	*Proteus vulgaris*	2	1.36	–	–	–
	*Providencia alcalifaciens*	1	–	–	0.68	–
*Aeromonadaceae* (8.16)	*Aeromonas caviae*	5	–	–	3.40	–
	*Aeromonas hydrophila*	3	–	–	2.04	–
	*Aeromonas veronii*	2	–	–	1.36	–
	*Aeromonas eucrenophila*	1	–	–	0.68	–
	*Aeromonas ichthiosmia*	1	–	–	0.68	–
*Erwiniaceae* (6.12)	*Pantoea agglomerans*	7	0.68	–	–	4.08
	*Erwinia* sp.	1	–	0.68	–	–
	*Erwinia persicina*	1	–	0.68	–	–
*Yersiniaceae* (2.72)	*Serratia proteamaculans*	1	–	–	–	0.68
	*Serratia grimesii*	1	–	–	–	0.68
	*Serratia fonticola*	1	–	0.68	–	–
	*Rahnella aquatilis*	1	–	0.68	–	–
*Staphylococcaceae* (2.72)	*Staphylococcus sciuri*	3	2.04	–	–	–
	*Staphylococcus epidermidis*	1	–	0.68	–	–
*Enterococcaceae* (2.04)	*Enterococcus asini*	2	–	–	1.36	–
	*Enterococcus faecium*	1	–	–	0.68	–
*Hafniaceae* (0.68)	*Hafnia alvei*	1	0.68	–	–	–
*Brucellaceae* (0.68)	*Ochrobactrum intermedium*	1	0.68	–	–	–
*Moraxellaceae* (0.68)	*Acinetobacter calcoaceticus*	1	–	–	–	0.68
Total	Isolates	147 (100%)	58.50	14.96	16.33	10.20
	Species (of which three identified to genus)	49 (100%)	40.82	28.57	26.53	16.33

^*a*^ – denotes lack of isolates.

**Fig 2:**
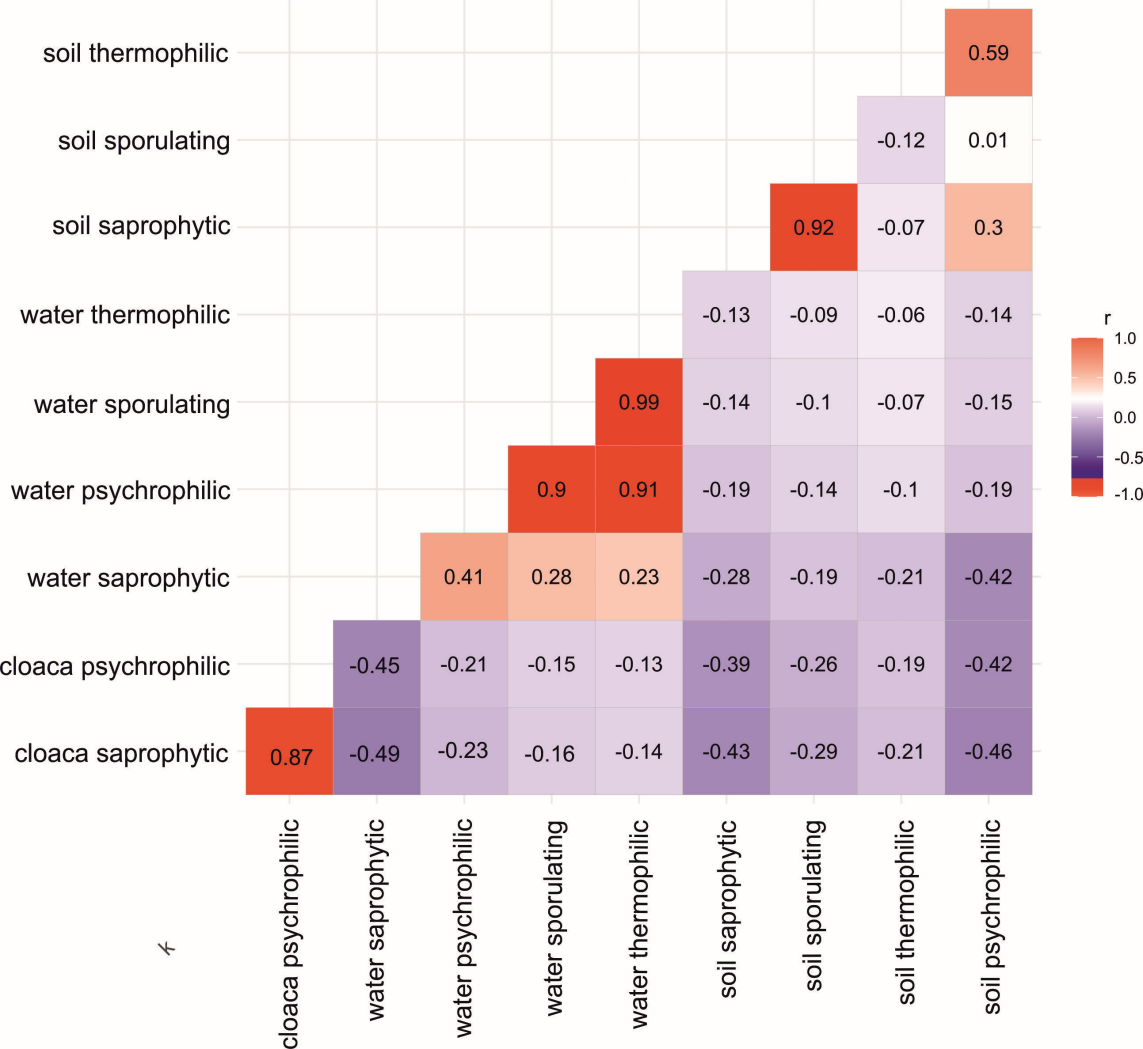
Correlation matrix showing Spearman rank correlation coefficients between selected functional groups of bacteria isolated from different environments (soil, water, and cloacal samples). The results indicate moderate negative relationships among certain bacterial groups, suggesting limited exchange or overlap in the microbial composition across these habitats.

Four main bacterial groups were identified in the environmental samples (soil and water): saprophytic, sporulating, thermophilic, and psychrophilic (Table 2). A Kruskal–Wallis test (*χ*² =49.60, df = 3, *P* < 0.001) showed significant overall differences in the abundance of these four groups, and Dunn’s *post hoc* analysis confirmed that each group differed from the others (*P* < 0.001 for all pairwise comparisons). When focusing on soil *vs* water, only sporulating bacteria varied significantly between these two environments (Mann–Whitney: W = 1635, *P* < 0.001), whereas saprophytic, thermophilic, and psychrophilic groups did not (*P* > 0.05 for each). By contrast, cloacal samples contained only saprophytic and psychrophilic bacteria, and these two groups differed substantially in their abundance (Mann–Whitney: W = 76, *P* < 0.001). Moreover, correlation coefficients (Fig. 2) indicated moderate negative relationships among certain bacterial groups in soil, water, and cloacal samples, suggesting a limited overlap or interchange in the microbial composition across these habitats.

**TABLE 2:** Total number of isolated microorganisms from water and soil of the nesting/feeding area of white stork^*a*^

Nesting/feeding area		Microbial count (CFU, colony-forming unit)				Total microbial count (CFU)
		Saprophytic	Sporulating	Thermophilic	Psychrophilic	
Brzezie Prawe (bp)	Water CFU·mL^−1^	3.2 × 10^5^	0	0	2.0 × 10^3^	3.22 × 10^5^
	Soil CFU·g^−1^	0	0	0	0	0
Brzezie Lewe (bl)	Water CFU·mL^−1^	3.2 × 10^4^	0	0	2.13 × 10^3^	3.41 × 10^4^
	Soil CFU·g^−1^	0	0	0	0	0
Chwalim (ch)	Water CFU·mL^−1^	2.4 × 10^4^	0	0	3.8 × 10^2^	2.44 × 10^4^
	Soil CFU·g^−1^	9.33 × 10^2^	5.33 × 10^1^	0	0	9.83 × 10^2^
Krężoły (k)	Water CFU·mL^−1^	2.13 × 10^3^	0	0	1.07 × 10^2^	2.24 × 10^3^
	Soil CFU·g^−1^	2.27 × 10^4^	8 × 10^1^	9.33 × 10^1^	3.33 × 10^5^	3.56 × 10^5^
Mieszkowo (m)	Water CFU·mL^−1^	0	0	0	0	0
	Soil CFU·g^−1^	2.27 × 10^3^	1.2 × 10^3^	0	2.53 × 10^3^	6 × 10^3^
Smolno Wielkie (sm)	Water CFU·mL^−1^	7.33 × 10^3^	0	0	5.07 × 10^2^	7.84 × 10^3^
	Soil CFU·g^−1^	3.2 × 10^4^	1.33 × 10^3^	0	1.11 × 10^5^	1.44 × 10^5^
Sulechów (s)	Water CFU·mL^−1^	2.93 × 10^5^	1.07 × 10^2^	0	0	2.93 × 10^5^
	Soil CFU·g^−1^	0	0	0	0	0
Swarzenice (swa)	Water CFU·mL^−1^	1.73 × 10^4^	0	0	0	1.73 × 10^4^
	Soil CFU·g^−1^	0	0	0	0	0
Wojnowo (w)	Water CFU·mL^−1^	1.6 × 10^3^	0	0	6.0 × 10^2^	2.2 × 10^3^
	Soil CFU·g^−1^	2.0 × 10^4^	1.47 × 10^3^	0	1.04 × 10^5^	1.25 × 10^5^
Gębice (g)	Water CFU·mL^−1^	2.93 × 10^4^	0	0	9.33 × 10^1^	2.94 × 10^4^
	Soil CFU·g^−1^	1.2 × 10^4^	1.47 × 10^3^	0	3.73 × 10^3^	1.72 × 10^4^
Włostów (wł)	Water CFU·mL^−1^	0	0	0	0	0
	Soil CFU·g^−1^	1.2 × 10^4^	6.67 × 10^2^	0	5.6 × 10^4^	6.87 × 10^4^
Kargowa (ka)	Water CFU·mL^−1^	3.33 × 10^3^	0	0	0	3.33 × 10^3^
	Soil CFU·g^−1^	1.6 × 10^4^	6.67 × 10^3^	0	5.47 × 10^3^	2.81 × 10^4^
Drągowina (d)	Water CFU·mL^−1^	3.47 × 10^4^	1.33 × 10^2^	2.13 × 10^3^	7.73 × 10^3^	4.47 × 10^4^
	Soil CFU·g^−1^	0	0	0	0	0
g, gr, kl, wic, lg, c	Water CFU·mL^−1^	0	0	0	0	0
	Soil CFU·g^−1^	0	0	0	0	0

^*a*^ c, Cieszów; g, Głuchów; gr, Górzykowo; kl, Klepina; lg, Leśna Góra; wic, Wicina.

### Local nest conditions and cloacal microbiota

To assess whether cloacal bacterial composition depended on nest-specific factors, a generalized linear mixed model (GLMM) was employed, with the number of isolated cloacal bacteria as the dependent variable, nest ID as a fixed effect, and the number of nestlings as a random factor. The results indicated a significant variation among nests (t = −5.53; *P* < 0.001; pseudo-R² =0.56), with no evidence that brood size (SD = 0.93) influenced the total count of cloacal bacteria. This finding suggests that local nest conditions shape the cloacal microbiota more strongly than the number of chicks in the nest. One could also suspect that the fact that the nest shapes the cloacal culturable bacteria may relate to the diet provided by the same parents as much as the nest environment.

### Antibiotic resistance

A total of 45 gram-negative bacterial species (140 isolates) and an additional 4 gram-positive species (seven isolates) were examined for antibiotic resistance. We tested susceptibility/resistance of all investigated isolates to all tested antibiotics, irrespective of the reported “intrinsic” resistance for some species. This approach was chosen because the “intrinsic resistance” is defined in relation to the clinical use of antibiotics, especially prevention of the use of chemotherapy during the infection event (47, 48). Thus, when most (or a large proportion) of strains of particular bacterial species are known to be resistant to the assessed antibiotic, there is no reason to use it in a clinical practice. However, this does not mean that all strains of such a bacterium reveal high levels of resistance; some susceptibility might be observed in a few strains. Therefore, when testing environmental samples, we wanted to avoid any overlooking of the resistance event.

**Fig 3:**
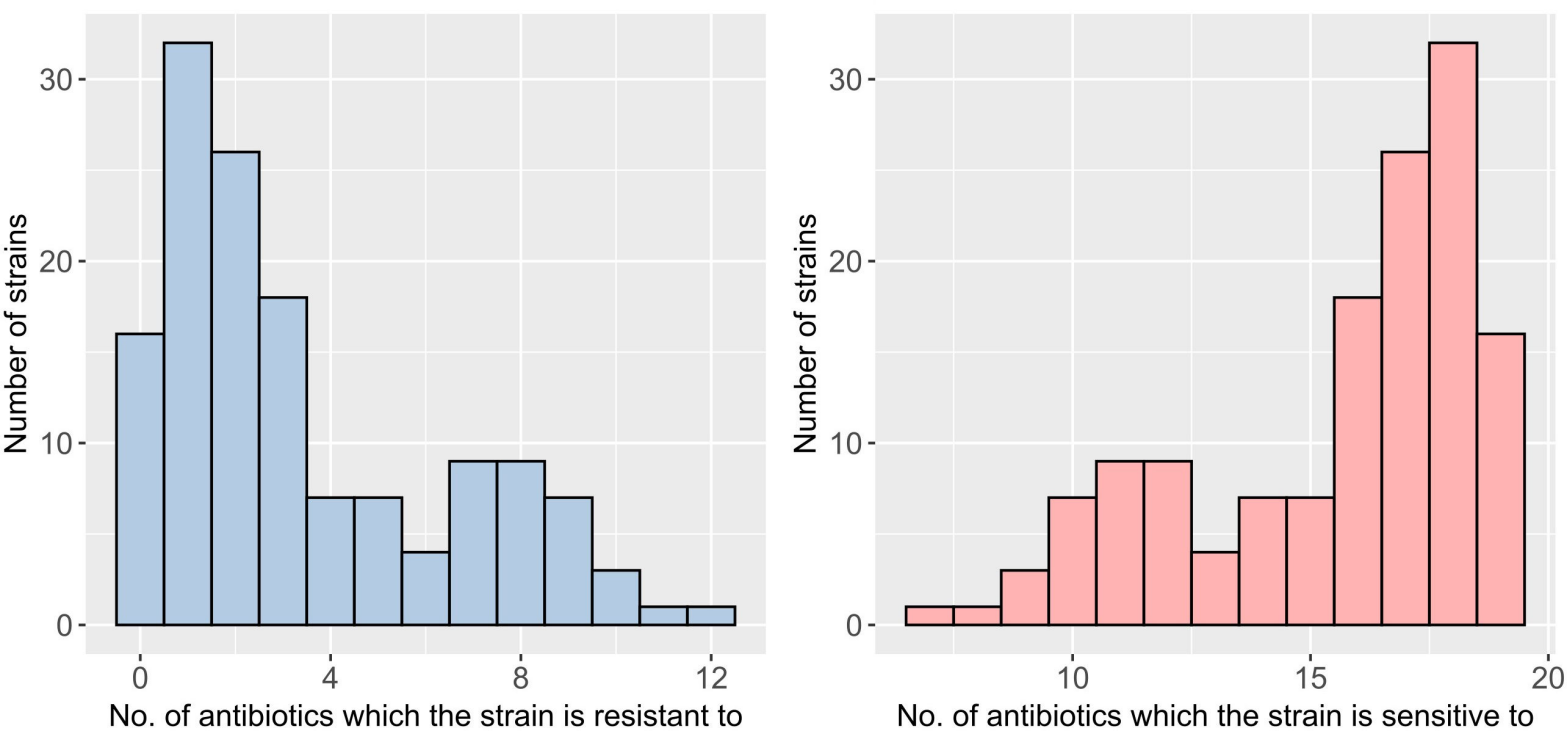
Distribution of strain numbers of gram-negative bacteria in relation to the 19 tested antibiotics (left panel—antibiotic resistance) and (right panel—antibiotic susceptibility).

Among gram-negative bacteria, the isolate resistant to the highest number of antimicrobial agents was *Ochrobactrum intermedium,* which displayed resistance to 12 of the 19 tested antibiotics in a single cloacal sample (Fig. 3). The mean number of antibiotics to which isolates were resistant was 4 (Fig. 3). Fully susceptible isolates, showing no resistance to any of the 19 antibiotics, included *Pseudomonas koreensis* (soil), *Pantoea agglomerans* (soil, cloaca), *Aeromonas caviae* (water), *Aeromonas hydrophila* (water), *Escherichia coli* (cloaca and one water isolate), *Enterobacter cloacae* (cloaca), *Serratia proteamaculans* (soil), and *Leclercia adecarboxylata* (cloaca) (Table 3).

**TABLE 3:** Prevalence and antibiotic resistance phenotypes and genotypes of bacteria isolated from white stork cloaca (*n* = 50), food (*n* = 16), water (*n* = 19), and soil (*n* = 19) of the foraging area^*a*^^,^^*b*^^,^^*c*^^,^^*d*^^,^^*e*^

Bacterial species (alphabetical order)	Prevalence (number of isolates)	Resistance to β-lactam antibiotics	Resistance to non-β-lactam antibiotics	Drug resistance genes		No. of drug-resistant isolates
				To β-lactams	To non-β-lactams	
*Acinetobacter calcoaceticus*	soil (1)	AM, TEM, CPZ, CAZ, CPM	NI	*bla*_CTX-M_ *, bla*_BIC-1_ *,* *bla*_SPM-1_ *,bla*_DIM-1_ *, bla*_KPC_ *, bla*_NDM_	*qnrB*	1
*Aeromonas caviae*	Water (5)	AM, SAM	ND	ND	ND	1
		AM, TEM	NI	ND	ND	1
		AM	ND	ND	ND	2
		ND	ND	*bla*_TEM_	ND	1
*Aeromonas eucrenophila*	Water (1)	AM, TC, SAM	ND	ND	ND	1
*Aeromonas hydrophila*	Water (3)	AM, TC, TEM, SAM, CAZ	S, ENF, NI	ND	*qnrS*	1
		AM, TC, SAM	ND	ND	ND	1
		ND	ND	ND	ND	1
*Aeromonas ichthiosmia*	Water (1)	AM, TC, SAM	ND	ND	ND	1
*Aeromonas veronii*	Water (2)	AM, TC, AUG, SAM, PTZ	ND	*bla*_CTX-M_	*qnrS*	1
		AM	ND	ND	ND	1
*Citrobacter amalonaticus*	Cloacal swab (1)	AM	CN	*bla*_TEM_	ND	1
*Citrobacter braakii*	Cloacal swab (1)	AM, AUG	ND	*bla*_CTX-M_ *, bla*_CMY-2_	ND	1
*Citrobacter freundii*	Cloacal swab (6)	AM, AUG	S, ENF, T	*bla*_CTX-M_ *, bla*_CMY-2_ *, bla*_NDM_	*qnrB*	2
		AM, AUG		*bla*_CMY-2_ *, bla*_VIM-2_ *, bla*_NDM_	ND	1
		AM, AUG	CN	*bla*_CMY-2_ *, bla*_VIM-2_	ND	1
		AM, AUG	ND	ND	ND	1
		ND	T	*bla*_CMY-2_ *, bla*_VIM-2_	ND	1
*Enterobacter cloacae*	Cloacal swab (6)	AM, AUG	ND	*bla*_BIC-1_	*qnrB*	1
		AM, AUG	ND	ND	ND	3
		AM, AUG	ND	*bla*_BIC-1_	ND	1
		ND	ND	ND	ND	1
*Enterococcus asini*	Water (2)	ND	CT	ND	ND	2
*Enterococcus faecium*	Water (1)	ND	CN, CIP, ENF, CT	ND	ND	1
*Erwinia persicina*	Food (1)	AM, TC, TEM, AUG, SAM, CAZ, CXT	NI	*bla*_NDM_	ND	1
*Erwinia* sp.	Food (1)	AM, TC	ND	ND	ND	1
*Escherichia coli*	Cloacal swab (35)	AM, TC, SAM	CN, CIP, ENF, TS, T	*bla*_TEM_	*qnrB*	1
		AM, TC	CIP, ENF	*bla*_TEM_ *bla*_VIM-2_*, bla*_DIM-1_*, bla*_KPC_	*qnrB*	1
		AM, TC	CN, CIP, ENF, T	*bla*_TEM_	*qnrB*	1
		AM,TC	NI	*bla_CTX-M_*	ND	1
		ND	CIP, ENF	*bla*_TEM_ *, bla*_VIM-2_	*qnrB*	1
		AM, TC	CN, S, TS	*bla*_TEM_ *, bla*_VIM-2_	ND	1
		ND	CN, CIP, ENF	ND	*qnrB*	1
		ND	CN, ENF	ND	ND	1
		ND	ND	*bla*_SPM-1_	ND	1
		ND	CN	ND	ND	1
		ND	ND	ND	ND	5
		ND	CN	ND	ND	20
	Water (2)	ND	ND	ND	ND	1
		AM, TC, SAM, CPZ	CN, S, CIP, ENF, TS	*bla*_TEM_	*aacC2, qnrB*	1
*Escherichia herman nii*	Cloacal swab (3)	AM, TC	NI	*bla*_CTX-M_ *, bla*_NDM_	*qnrB*	1
		AM, TC	NI	ND	ND	1
		AM, TC	NI	ND	*aacC2*, *qnrB*	1
*Escherichia vulneris*	Cloacal swab (1)	AM, TC	CN	*bla*_TEM_ *, bla*_VIM-2_	ND	1
*Hafnia alvei*	Cloacal swab (1)	AM, AUG, SAM	ND	*bla*_VIM-2_, *bla*_DIM-1_*, bla*_NDM_	ND	1
*Klebsiella oxytoca*	Water (2)	AM, TC, CAZ	CIP, ENF	*bla*_CTX-M_ *, bla*_SHV_	*qnrB, qnrS*	1
		AM, TC	CIP, ENF	ND	*qnrB, qnrS*	1
*Klebsiella pneumoniae*	Water (2)	AM, TC	T	*bla*_SHV_	ND	1
		AM, TC	ND	*bla*_SHV_	ND	1
*Klebsiella* sp.	Food (1)	AM, TC	ND	*bla*_CTX-M,_ *bla*_BIC-1_ *, bla*_NDM_	*qnrB*	1
*Leclercia adecarboxylata*	Cloacal swab (1)	ND	ND	*bla*_CTX-M_ *, bla*_VIM-2_ *, bla*_DIM-1_	ND	1
*Ochrobactrum intermedium*	Cloacal swab (1)	AM, TC, TEM, AUG, SAM, PTZ, CAZ, CTX, CPM	NI, T, CT	*bla*_SHV_ *, bla*_NDM_	ND	1
*Pantoea agglomerans*	Cloacal swab (1)	ND	ND	*bla*_SHV_ *, bla*_VIM-2_ *, bla*_NDM_	ND	1
	Soil (6)	AM	ND	*bla*_VIM-2_ *,bla*_NDM_	*qnrB*	1
		AM, TC	T	*bla*_CTX-M_ *, bla*_VIM-2_	ND	1
		AM	ND	ND	ND	1
		ND	ND	*bla*_VIM-2_ *, bla*_NDM_	ND	1
		ND	ND	*bla*_NDM_	ND	1
		AM, TC	ND	ND	ND	1
*Proteus mirabilis*	Cloacal swab (11)	AM, TC	CIP, ENF, NI, TS, T, CT	*bla*_TEM_	ND	1
		ND	CT	*bla*_TEM_	ND	1
		AM	TS, T, CT	*bla*_TEM_ *, bla*_OXA-48_	ND	1
		ND	ENF, T, CT	ND	ND	1
		AM, TC,	ENF, NI, TS, T, CT	*bla*_TEM_ *, bla*_OXA-48_	ND	1
		ND	T, CT	ND	ND	1
		AM, TC, SAM	CN, CIP, ENF, NI, TS, T, CT	*bla*_OXA-48_	ND	1
		ND	AK, CN, ENF, NI, TS, T, CT	ND	ND	1
		AM, AUG, SAM	CN, CIP, ENF, NI, T, CT	*bla*_TEM_	ND	1
		AM, TC	CIP, ENF, NI, TS, CT	*bla*_TEM_	*qnrB*	1
		AM	AK,CN,S,ENF,T,CT	*bla*_TEM_	ND	1
	Water (3)	AM, CPZ	CT	ND	ND	1
		AM	CT	ND	ND	2
*Proteus vulgaris*	Cloacal swab (2)	AM	NI, CT	ND	*qnrB*	1
		AM	NI, T, CT	ND	ND	1
*Providencia alcalifaciens*	Cloacal swab (1)	ND	NI, T, CT	*bla*_SHV_ *, bla*_DIM-1_	ND	1
*Pseudomonas aeruginosa*	Cloacal swab (4)	AM, TEM, AUG, SAM, CTX	ENF, NI, T, TS	*bla*_TEM_, *bla*_NDM_	ND	1
		AM, TEM, AUG, SAM, CTX	ENF, NI, T, TS	*bla*_SPM-1_ *, bla*_NDM_	*qnrB*	1
		AM, TEM, AUG, SAM, CTX	ENF, NI, T, TS	*bla*_SPM-1_ *, bla*_KPC_	ND	1
		AM, TEM, AUG, SAM, CTX	ENF, NI, T	*bla*_KPC_	ND	1

*Pseudomonas antarctica*	Food (1)	AM, TC, TEM, AUG, SAM, CAZ, CTX	NI	ND	ND	1
*Pseudomonas corrugata*	Food (2)	AM, TC, TEM, SAM	ENF, NI	*bla*_SHV_, *bla*_NDM_	ND	1
		AM, TC, SAM	NI	ND	ND	1
*Pseudomonas extremorientalis*	Food (1)	AM, TC, CAZ, CPM, CTX, AUG, SAM	NI	*bla*_TEM_	ND	1
*Pseudomonas flavescens*	Soil (1)	AM	NI	*bla*_BIC-1_	*qnrB*	1
	Cloacal swab (3)	AM	NI	ND	ND	2
		AM	NI	*bla*_TEM_	ND	1
						
*Pseudomonas frederiksbergensis*	Food (1)	AM, TC, TEM, AUG, SAM, CTX	NI	*bla*_SPM_ *, bla*_DIM-1_	ND	1
*Pseudomonas fulva*	Cloacal swab (2)	AM, TC, TEM, AUG, SAM, CTX	ENF, NI, T	*bla*_TEM_	ND	1
		AM, TC, TEM, AUG, SAM, CTX	ENF, NI, T	ND	ND	1
*Pseudomonas jessenii*	Food (3)	AM, TEM, TC, AUG, SAM, CTX	ND	*bla*_CTX-M_	ND	1
		AM, TEM, TC, AUG, SAM, CTX, CPM, CAZ	TS, NI	ND	ND	1
		AM, TEM, TC, AUG, SAM, CTX	NI	ND	ND	1
*Pseudomonas koreensis*	Soil (1)	ND	ND	ND	*qnrB*	1
	Food (5)	AM, SAM, CPZ	NI	*bla*_TEM_ *, bla*_OXA-48_ *, bla*_SPM-1,_ *bla*_VIM-2_	*qnrB*	1
		AM, TC, CPZ	ND	*bla*_CTX-M_	ND	1
		AM, TC, TEM, AUG, SAM, CTX	NI	*bla*_VIM-2_ *, bla*_NDM_	ND	1
		AM, TC, TEM, AUG, SAM, CTX, CAZ	NI	*bla*_SHV_	ND	1
		AM, TC, TEM, AUG, SAM, CTX, CAZ	NI	*bla*_CTX-M_ *, bla*_VIM-2_ *, bla*_NDM_	*qrnA*	1
*Pseudomonas putida*	Water (1)	AM, TC, TEM, CPZ, CTX	ENF, TS, NI, T	*bla*_CTX-M_	ND	1
*Pseudomonas synxantha*	Food (1)	AM, TC, TEM, CAZ, AUG, SAM, CTX	NI	*bla*_NDM_	ND	1
*Pseudomonas thivervalensis*	Food (2)	AM,TEM,TC, CPZ, SAM,	CIP, TS, ENF, NI,	ND	*qnrA*	1
		AM, TEM, TC, SAM, AUG, CXM	NI	ND	ND	1
*Pseudomonas* sp.	Cloacal swab (1)	AM, TEM, CAZ, CTX,	CT	ND	ND	1
*Rahnella aquatilis*	Food (1)	AM, TC, CPZ, CTX	T	ND	ND	1
*Raoultella ornithinolytica*	Cloacal swab	AM, TC	ND	ND	ND	1
	Soil (1)	AM	ND	ND	ND	1
*Raoultella planticola*	Food (1)	AM, TC, CZ, KF, CXM, CPD, CPZ, CTX	T	ND	ND	1
*Salmonella enterica*	Cloacal swab (2)	ND	CN	*bla*_NDM_	ND	2
*Serratia fonticola*	Food (1)	AM,TC	ND	*bla*_CTX-M_ *, bla*_TEM_ *, bla*_SPM-1_ *, bla*_VIM-2_ *, bla*_DIM-1_	*qnrA*	1
*Serratia grimesii*	Soil (1)	AUG	NI	*bla*_VIM-2_	ND	1
*Serratia proteamaculans*	Soil (1)	ND	ND	ND	ND	1
*Staphylococcus epidermidis*	Food (1)	ND	CT	ND	ND	1
*Staphylococcus sciuri*	Cloacal swab	ND	CN, CT	ND	ND	1
		ND	CT	ND	ND	2
Isolates	147					147
Species (including three isolates identified to the level of genus)	49					49

^*a*^ General: ND, not detected.

^*b*^ β-lactam antibiotics: penicillins: AM, ampicyline; TC, ticarcillin; TEM, temocillin; P, penicillin (for gram-positive cocci); penicillins with inhibitor: SAM, ampicyline-sulbactam; AUG, amoxicillin-clavulanic acid; PTZ, piperacillin-tazobactam; third-generation cephalosporins: CPZ, cefoperazone; CAZ, ceftazidime; CTX, cefotaxime; fourth-generation cephalosporins: CPM, cefepime.

^*c*^ Non-β-lactam antibiotics: aminoglycosides: AK, amikacin; CN, gentamicin; S, streptomycin; quinolones: CIP, ciprofloxacin; ENF, enrofloxacin; chemotherapeutic agents: NI, nitrofurantoin; TS, trimethoprim-sulfamethoxazole; T, tetracycline; CT, colistin.

^*d*^ Drug resistance genes to: extended-spectrum β-lactams (ESBL): *bla*_CTX-M_, CTX-M; *bla*_SHV_, SHV; *bla*_TEM_, TEM; *bla*_CMY-2_, CMY-2; carbapenems (CPs): *bla*_OXA48_, OXA-48; *bla*_BIC-1_, BIC-1; *bla*_SPM-1_, SPM-1; *bla*_VIM-2_, VIM-2; *bla*_DIM-1_, DIM-1; *bla*_KPC_, KPC; *bla*_NDM_, NDM; aminoglycosides (AMG): *aacC2*; quinolones (Q): *qnrA*; *qnrB*,* qnrS*.

^*e*^ The antibiotic resistance was determined according to (49).

**Fig 4:**
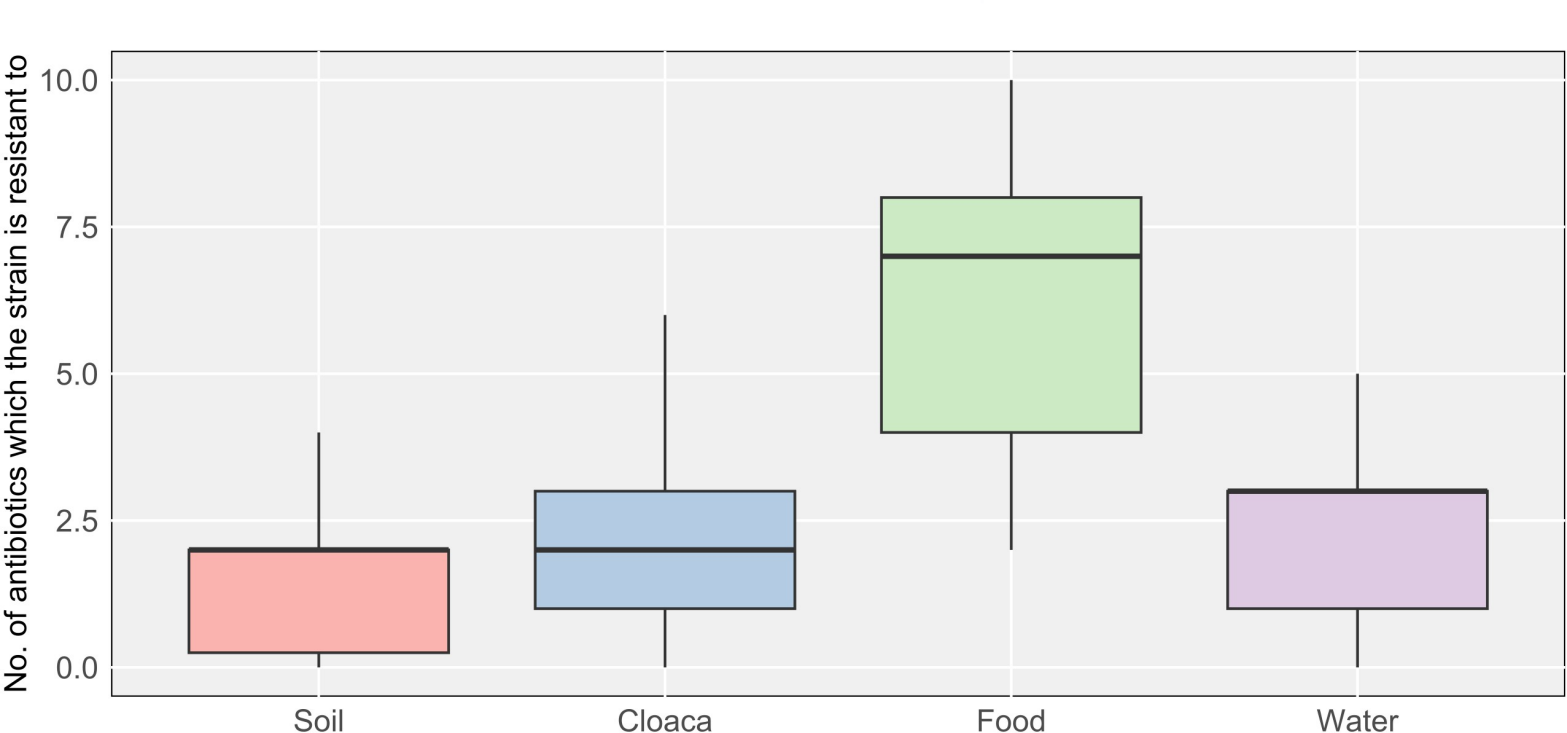
Differences in the number of antibiotic-resistant strains among the four bacterial groups. The Kruskal–Wallis test (*χ*² = 23.004; df = 3; *P* < 0.001) revealed significant variation in resistance depending on sample origin (food, soil, cloaca, and water), with food-derived isolates showing the highest resistance. Dunn’s *post hoc* test confirmed significant differences between food-derived isolates and those from soil (*P* < 0.001), cloaca (*P* < 0.0001), and water (*P* < 0.009), while no significant differences were observed among the latter three groups (*P* > 0.05).

A Kruskal–Wallis test (*χ*² =23.004; df = 3; *P* < 0.001) showed that the overall resistance of gram-negative isolates varied significantly depending on sample origin (cloaca, food, water, and soil). Dunn’s *post hoc* test indicated that food-derived isolates were the most resistant, differing significantly from those found in soil (*P* < 0.001), cloaca (*P* < 0.0001), and water (*P* < 0.009). No significant differences in resistance emerged among isolates from soil, water, and cloaca (*P* > 0.5) (Fig. 4).

**Fig 5:**
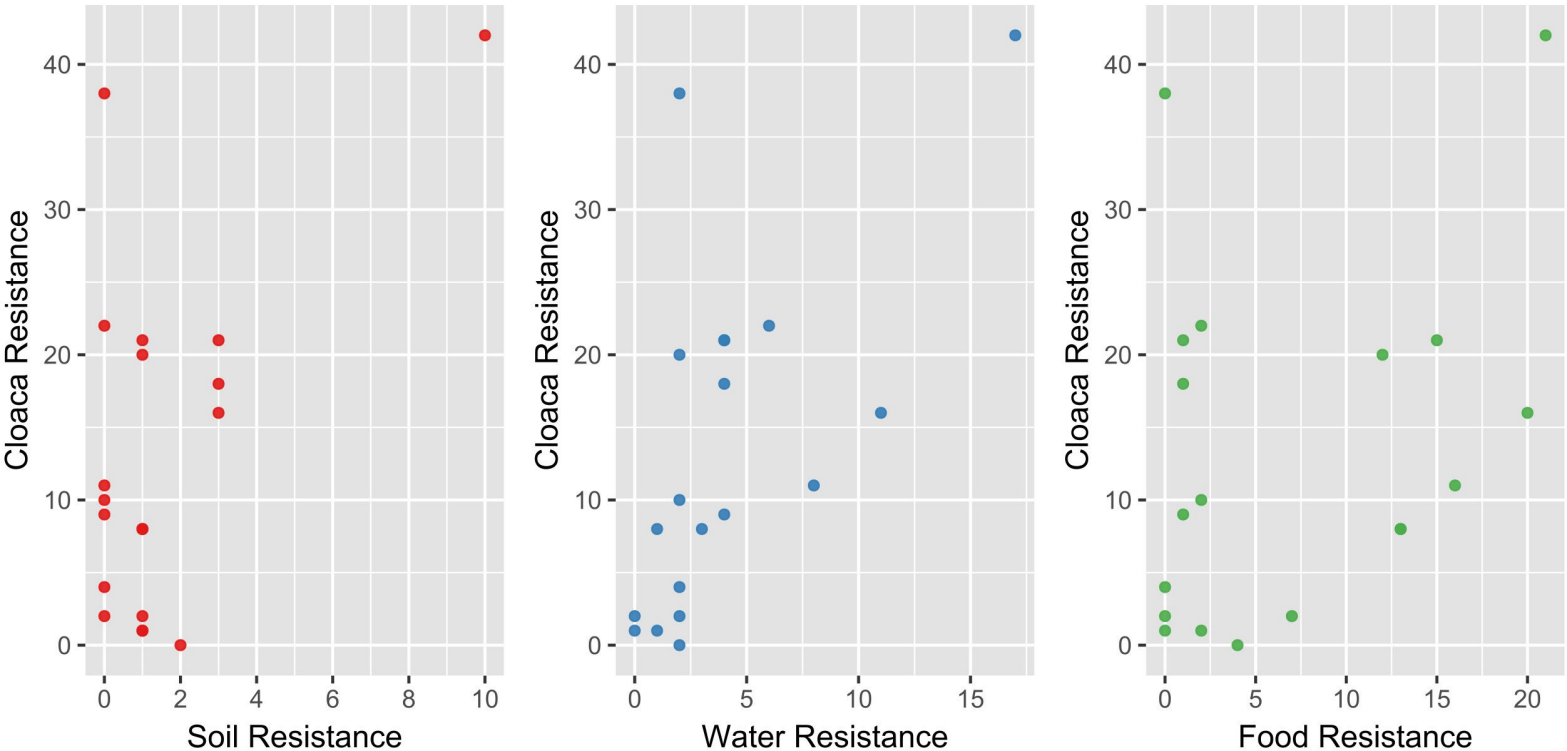
Relationships between antibiotic resistance profiles of gram-negative bacterial isolates from cloacal samples and those from soil, water, and food sources. Spearman’s rank correlations based on the number of antibiotics to which each isolate was resistant, indicating no significant correlation between cloaca and soil (rs = 0.11; *P* = 0.647) or cloaca and food (rs = 0.25; *P* = 0.289). However, a significant correlation was observed between cloacal and water isolates (rs = 0.68; *P* = 0.001).

Based on the number of antibiotics to which each isolate was resistant, Spearman’s rank correlations were used to compare profiles between cloacal and environmental samples. No significant correlations were found between cloaca and soil (rs = 0.11; *P* = 0.647) or between cloaca and food (rs = 0.25; *P* = 0.289), whereas cloacal and water isolates showed a correlation (rs = 0.68; *P* = 0.001) (Fig. 5). To perform a deeper analysis of this pattern, antibiotics were grouped into functional classes: penicillins, penicillins with β-lactamase inhibitors, third-generation cephalosporins, fourth-generation cephalosporins, aminoglycosides, quinolones, chemotherapeutic agents, tetracyclines, and polymyxins. A similar pattern was observed; there was no significant correlation between resistance profiles in cloacal and soil isolates (rs = 0.001, *P* = 0.999) or between cloacal and food isolates (rs = 0.26, *P* = 0.484), while a significant correlation remained between cloacal and water isolates (rs = 0.67, *P* = 0.047). These findings indicate that antibiotic resistance profiles in cloacal isolates resemble more closely those found in waterborne bacteria than in bacteria from food or soil, even when resistance is analyzed by the antibiotic class. This similarity may reflect shared environmental exposure rather than direct transmission.

**Fig 6:**
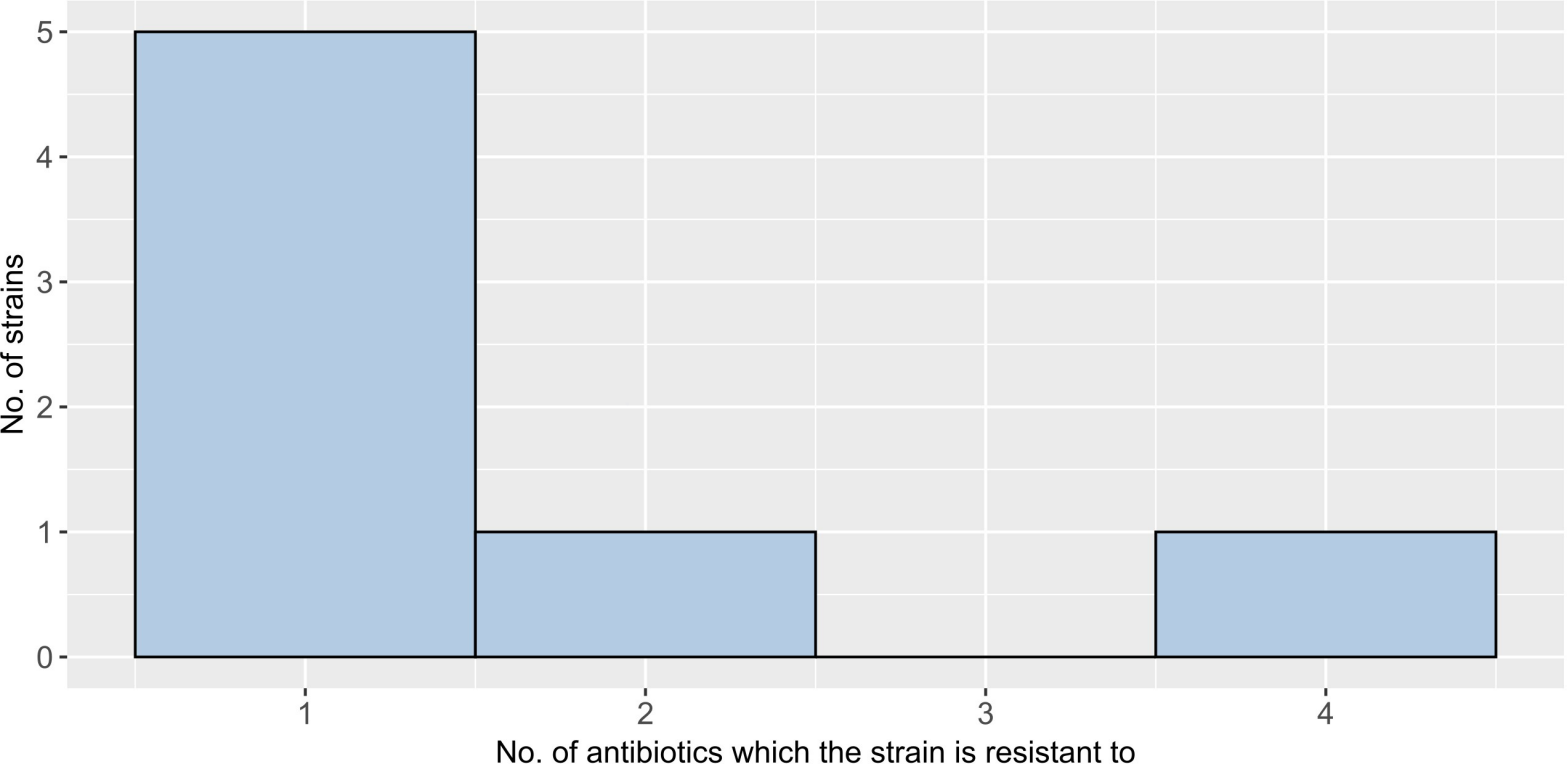
Distribution of drug resistance in four species of gram-positive bacteria from seven isolates.

Among gram-positive bacteria (seven isolates), four species were detected: *Staphylococcus epidermidis* (food*), Staphylococcus sciuri* (cloaca), *Enterococcus faecium* (water), and *Enterococcus asini* (water). None of these isolates were susceptible to all 19 antibiotics (Fig. 6). The highest resistance was observed in *Enterococcus faecium*, which was resistant to four antibiotics in a single water isolate. A Mann-Whitney test (W = 4.0; *P* = 0.99) comparing gram-positive bacteria from cloacal versus water samples showed no significant difference in their resistance levels.

Among the 147 isolates (gram-negative and gram-positive species), specific antibiotic resistance genes (responsible for resistance to either β-lactam or non- β-lactam antibiotics) were detected in 71 of them (Table 3). However, many isolates revealed the resistance phenotype despite the lack of tested antibiotic-resistance genes (perhaps they did bear other genes or were intrinsically resistant to the investigated antibiotics), while some were susceptible to all antimicrobial agents from the tested group (β-lactam or non-β-lactam compounds) despite the presence of specific DNA sequences indicating the presence of antibiotic-resistance genes. Examples are *Pantoea agglomerans* isolates 4 and 5, bearing either *bla*_VIM-2_ and *bla*_NDM_, or *bla*_NDM_ alone, and *Providencia alcalifaciens* bearing *bla*_SHV_ and *bla*_DIM-1_ genes, while being sensitive to all tested β-lactam antibiotics. Moreover, *Enterobacter cloacae* isolate 1, *Klebsiella* sp., *Pantoea agglomerans* soil isolate 2, and *Pseudomonas koreensis* soil isolate contained the *qnrB*-specific DNA sequence, and the presence of the *qnrA* sequence was detected in the *Seratia fonticola* genome, whereas all these isolates were sensitive to all tested non-β-lactam antibiotics (Table 3). Potential explanations of the susceptibility to antibiotics despite the presence of DNA sequences characteristic for antibiotic-resistance genes could be either mutations inactivating the specific gene products or a lack of expression of these genes due to either mutations in the regulatory sequences or negative regulation of the gene expression under certain growth conditions.

## DISCUSSION

The crucial discovery presented in this paper is that while a correlation was observed between cloacal isolates and those from water, no similar relationship was found with soil or the stork’s food. Notably, the studied area encompasses both agricultural and industrial/urban environments. Although some bacteria from water and cloaca shared resistance to similar antibiotics, no evidence was found for the identical bacterial isolates (in terms of the same resistance pattern and antibiotic-resistance gene(s)) occurring in both a cloacal sample and any other tested habitat (Table 3). Consequently, these findings suggest that white storks may play a less significant role in transmitting antibiotic-resistant bacteria than it might be assumed for a synanthropic species frequently foraging in farmland and near rivers (50). One might assume that bacteria acquired through food from agricultural and urban sources may not readily persist within a stork’s gastrointestinal tract, possibly due to competitive interactions with the resident microbiota (this restriction might be less pronounced in the case of water sources, for as yet an unknown reason). While this does not rule out occasional horizontal transfer of antibiotic resistance genes, in the absence of a strong selective pressure within the bird’s body, stable maintenance of such genes in acquired bacteria appears to be relatively uncommon. Thus, even with the correlation observed in water-borne isolates, the overall scenario of storks facilitating large-scale transmission of antibiotic resistance between different environments appears more constrained than one might expect based on their synanthropic behavior.

The above suggestion indicates that previous hypotheses on the role of wild birds as vectors in the global spread of antibiotic-resistant bacteria should be reconsidered (27–31). There are numerous examples of antibiotic-resistant bacteria in biological material derived from wild birds (32–34). There is no doubt that these bacteria may be a source of antibiotic-resistance genes in the environment when excreted with feces. However, antibiotic resistance is a naturally occurring phenomenon, and the presence of antibiotic-resistant bacteria is not necessarily directly related to humans’ use of antibiotics. Indeed, the presence of antibiotic resistance genes was reported in environmental samples as old as 13,000 years, where no influence of human civilization was possible (51). Therefore, the role of wild birds in transmission of antibiotic-resistant bacteria appears not obvious and requires further consideration, perhaps with re-examining this phenomenon using more precise monitoring methods.

It should also be noted that the mixed model results indicated that bacterial flora differed significantly among nests, while no significant differences were found within individual broods. This suggests that local environmental conditions play a dominant role in shaping the cloacal microbiota. This finding implies that each breeding pair likely exploits distinct foraging territories and food sources, leading to unique bacterial communities in nestlings. White storks can exhibit strong site fidelity or territorial behavior around their nests, which could limit overlap in the environments each brood is exposed to (52). Consequently, differences in water bodies, agricultural fields, or other feeding habitats near each nest may be driving the observed variation in cloacal bacterial profiles.

Apart from the main conclusion presented and discussed above, this study led to some other, perhaps quite important observations, expanding our understanding of the environmental biodiversity of antibiotic-resistant bacteria and the general composition of bacterial species. The most abundant bacterial group in both environments (stork cloaca and soil/water/food) was saprophytes, followed by psychrophilic and sporulating bacteria. Only in one sample of water and soil were thermophilic bacteria found. The highest number of microorganisms was found in three locations (Brzezie Prawe, Drągowina, and Sulechów) for water samples and in three locations (Krężoły, Smolno Wielkie, and Wojnowo) for soil samples, while the lowest total number of microorganisms was determined at a single location (Mieszkowo) (Table 2). The results obtained in this work regarding bacterial growth indicate low bacterial abundance. The values obtained for sporulating forms (January, February, and March) were higher by an order of magnitude up to two, and for October and November by as much as 2 × 10^3^; however, similar values were obtained for vegetative forms for only three locations (Krężoły, Smolno Wielkie, and Wojnowo) as in October and the first half of November (52).

As previously presented (53), *Aeromonas* spp. found in aquatic environments contains many species that are pathogenic, mainly to fish, including those presented in this report. Along with many other species, the importance of bacteria to plants, including *Pantoea agglomerans* (54) and *Acinetobacter calcoaceticus* (55), was indicated. Bacteria were cultured from earthworms and spider worms (food) collected from the white stork nesting/feeding area. Twenty-two isolates were obtained, representing about 15% of all isolates. Fourteen species were identified. Of all the isolated species, 13 were obtained from food alone, of which the genus *Pseudomonas* was the most abundant (seven species) and two species each from the genus *Erwinia* and the family *Yersiniaceae* (Table 3). The species identification results obtained here are similar to those described previously (56, 57).

The results obtained here for identifying isolates from white stork cloaca in terms of family or genus are similar to those presented recently (58), obtained on the basis of analyses of 16S rRNA sequences. Other studies from the same geographical region (Poland) demonstrated that in samples derived from white storks, there were bacterial strains belonging to *Enterococcus faecalis, Staphylococcus aureus, Pseudomonas* sp. (24), *Campylobacter jejuni* (59), *Proteus mirabilis* (60), *Salmonella* sp. (61), *Gemmobacter intermedius* (62), *Corynebacterium tracheae* (63), *Corynebacterium pelargi* (64), and *Psychrobacter ciconiae* (65). Some isolates from our work also confirmed the presence of bacterial species mentioned in those previous studies.

It was observed that only *Escherichia coli* strains were devoid of resistance to non-β-lactam drugs in wildlife in Catalonia (6). However, in our collection, such isolates belonged to *Pantoea agglomerans* (six strains), *Pseudomonas flavescens* (four strains), *Pseudomonas koreensis* (five strains), *Leclercia adecarboxylata* (one strain), *Aeromonas caviae* (five strains), *Serratia proteamaculans* (one strain), *Escherichia coli* (three strains), and *Enterobacter cloacae* (six strains). They were isolated from soil (seven isolates), cloacal swabs (10 isolates), and water (five isolates). The occurrence of resistance to antibiotics from only the β-lactam or non-β-lactam antibiotic group was also reported (6–8). A higher proportion of strains resistant to β-lactam versus non-β-lactam antibiotics was noted (6), while in another study, no difference in antibiotic resistance among strains in this regard was observed (8). It can, therefore, be assumed that antibiotic sensitivity of bacterial strains is a manifestation of the microbial response to environmental factors. In such cases, it is the presence of antibiotics that generates conditions in the environment, meaning their habitat, and the bacteria must adapt to survive. In the presence of antibiotics in the environment, multi-sensitive strains became less frequent as they were eliminated under the selective pressure. Otherwise, in the lack of antibiotics, sensitive strains would have an advantage and might outgrow the resistant ones, which have a ballast of the antibiotic-resistance genes or reveal some small structural or metabolic defects, allowing the resistance but being useless in the absence of antimicrobial agents. In this work, for more than half of the tested strains (56.46%), no resistance genes to β-lactam antibiotics (ESBL and CPs) were found. In the case of resistance genes to non-β-lactam antibiotics, they were not found in most of the isolates studied (83%). The area of Poland is generally considered as having a relatively low level of resistance presence among strains; however, a higher share of resistance to β-lactams than to quinolones among strains was evident, while there is an increasing tendency for the emergence of strains resistant to the second-generation quinolones (6–8). Of all isolated species, drug resistance analyzed from the non-β-lactam antibiotic group was most widely presented in *Escherichia coli* and *Proteus mirabilis* isolates from cloacal swabs.

Some limitations of this study should be noted. First, the nests included in this study were located relatively close to one another (Fig. 1), which may reduce the ecological contrast between their respective foraging territories and influence the similarity of their resistance profile. Second, because our approach was correlational rather than experimental, we could not establish direct cause-and-effect relationships but only associations between bacterial isolates, habitat use, and antibiotic resistance patterns. Third, in studies involving relatively small sample sizes, even one outlier or unusual data point can significantly influence statistical significance, as seen in the water–cloaca correlation for antibiotic resistance (see Fig. 5). In fact, without the single data point seen in the panel’s upper-right corner, no significant correlation could be found in this case (similarly to the results obtained in testing soil-cloaca and food-cloaca analyses of antibiotic resistance, where no significant correlations were detected). Such sensitivity to individual measurements underscores the need for cautious interpretation of these results. It highlights the importance of increasing sample sizes and using additional methods (like experimental approaches) in future research. Fourth, all data in this study were collected during a single breeding season (2019), which limits the ability to assess the temporal variation in environmental conditions, bacterial communities, and resistance patterns. As such, the generalizability of the observed relationships in a long-term perspective remains uncertain. Repeating this approach over multiple breeding seasons would allow stronger inferences about the consistency and potential drivers of antibiotic resistance dynamics in white stork populations. Fifth, in this work, cloacal swabs were used for analyses of bacteria present in storks, while on the basis of analyses of microorganisms isolated from different materials derived from juvenile ostriches (*Struthio camelus*), it was concluded that sampling feces might provide more accurate assessment of the colon microbiome than sampling cloacal swabs (66). However, we have chosen to sample cloacal swabs rather than feces as the aim of this work was assessment of antibiotic resistance of bacteria rather than analysis of the colon microbiome. In the case of testing the presence of antibiotic resistance, we were afraid of possible contamination of feces with bacteria derived from the environment as it would not be technically possible to withdraw a sample of only very fresh feces derived from wild birds. While in investigations of the colon microbiome a negligible contamination of samples with environmental bacteria would perhaps be a little problem, then with testing antibiotic resistance, any contamination could significantly influence the results of analyzes, especially due to selective conditions in subsequent steps of the experiments. Therefore, we decided to collect cloacal swabs to be sure that all isolated bacteria derive from the birds, not being environmental contamination.

## MATERIALS AND METHODS

### Study area

The study was conducted in the Zielona Góra district in western Poland. The medium-sized city of Zielona Góra (51.933230, 15.518667), which has approximately 140,000 inhabitants, is located in the center of the study area. We examined 19 nests situated in the rural surroundings of the city (Fig. 1). All samples were collected within a 1.5 km radius of each nest. That distance is defined as the potential foraging range of storks from the nest, in population on the Western part of Poland and Europe (67). The landscape in this area is mainly mosaic arable fields, forests, farm buildings, meadows, and pastures. Watercourses or water reservoirs occur in the foraging areas of each studied nest and can be found within a 1.5 km radius of the nest. According to the Corine Land Cover 2012 system, arable land, forests, and pastures cover 52%, 22%, and 11% of the area, respectively. Water bodies occupy only 2% of the study area. All study nests occur near larger rivers. The Barycz River flows via 3 out of 19 foraging areas near Sulechów (52.086228; 15.622350). The Bóbr River flows across 2 out of 19 foraging areas near Nowogród Bobrzański (51.799457; 15.237486). The Odra River is the biggest river in this area, but it flows only through one of the foraging areas. As indicated in Fig. 1, Sulechów is the town closest to investigated nests; however, it is located over 10 km away from the nearest nest. Nevertheless, there are several small villages (listed in Table 2) around the investigated nests, which can be considered point sources of bacteria.

### Sample collection

Cloacal samples were taken from 50 white stork nestlings in 19 nests (Fig. 1) during the ringing procedure at the end of June 2019, when the nestlings were on average 38.8 ± 12.2 (SD) days old, and environmental samples were collected from January to November (Fig. 1). Swab sampling happened during the ringing campaign, but over time, the idea emerged to enrich the collection with environmental samples. Based on ornithological observations and literature data, points within a 1.5 km radius of the nest were designated (40, 68–70).

Swabs were immediately transferred to Amies transport medium and stored at 4°C until direct plating. Environmental samples were stored at 4°C for further analysis. After collection, samples were delivered to the laboratory within 24 hours. A new set of disposable gloves was used at each nest and at every environmental sampling site. Samples were immediately transported to the laboratory. The collected swabs and environmental samples were stored from their acquisition until analysis at 4°C, which was ensured by using a cooling container. Collection of stork samples was approved by the Local Ethics Committee for Experiments on Animals in Wrocław (decision no. 47/2017). The sterile agar gel medium transport swabs were used. Samples from the foraging area contained the following: (i) water from water reservoirs (small lakes, ponds, and small water courses); (ii) soil, and (iii) invertebrates, which were considered potential food for nestlings (Fig. 1).

The foraging sites were known to be actively used by adult white storks from the studied population. The selection of the sampling sites was based on direct field observations and local knowledge of stork foraging behavior. These locations were situated within a 1.5 km radius of the respective nests, corresponding to the typical foraging range of white storks during the breeding season (50, 67).

Nineteen water and soil samples and 16 stork food samples were collected. Fourteen food samples contained at least three earthworms (Lumbricidae), two samples contained at least two earthworms and two spider worms each. These invertebrates represent prey items commonly observed in white stork chick diet, particularly during early and mid-nestling stages (40, 68).

### Physicochemical properties of the water and soil samples

The soil pH was determined by the colorimetric method using a Hellig acid meter (approximate measurement). The cavity in the porcelain Hellig plate was filled with soil, which was then combined with Hellig’s liquid until the soil was completely wetted and a thin layer of liquid formed above it. After about 2–3 min, the color of the fluid in the channel of the plate was compared with the pH scale on the plate corresponding to a specific pH value (71). The pH of the water was determined using a pH meter. The measurement was performed three times, and the final result was the mean value of three measurements, calculated with an accuracy of 0.05 pH unit (72).

To indicate the physicochemical conditions of soil and water samples tested microbiologically, their pH values were determined. The pH tests of the collected soil samples showed that they were acidic or slightly acidic (12/19), and the remaining soils were strongly acidic (1/19), neutral (4/19), or alkaline (2/19) (Table S1). These results classify these soils as typical of the area, according to previously reported results (73). The pH values for the water samples were between 7.08 and 7.76 (17/19). The 6.95, 7.99, and 8.26 pH values were determined only in three water samples.

### Microbiological analyses

Standard microbiological methods were used to isolate and purify bacterial strains. In total, 50 samples of cloacal swabs, 19 water samples, 19 soil samples, and 16 food samples were collected. The total number of bacterial cells, and groups of saprophytic, psychrophilic, and thermophilic bacteria were determined on enriched agar (Graso Biotech, Poland) from the collected cloacal swabs, water, and soil samples. Ten grams of soil was suspended in 0.85% NaCl at the 1:9 wt/vol ratio. Dilutions from 10^−1^ to 10^−4^ were made for water and soil samples, and 0.25 mL of each dilution was spread over the entire surface of a Petri dish (φ 90 mm) with a sterile spatula. To assess the number of cells of sporulating bacteria, the prepared dilutions were incubated at 80°C for 15 min. Plates were incubated at 20°C (for psychrophilic species) or 28°C (for saprophytic and sporulating microorganisms) for 48 h or at 55°C (thermophilic species) for 24 h. Results are given as colony-forming units (CFU) per 1 g of soil or 1 mL of water. Each measurement was performed in triplicate. The collected food samples were crushed while maintaining sterility, and the material with a sterile swab (Biomedico) was applied to plates with nutrient medium (Columbia Agar with 5% blood, MacConkey agar, CHROM agar Salmonella Plus; Graso Biotech, Poland). Plates were incubated at 35±2°C for 24 h or 30°C for 48–72 h. White stork cloacal swabs in AMIES transport medium (Deltalab, Barcelona, Spain) and collected water and soil samples were plated on solid medium (Columbia Agar with 5% blood, MacConkey agar, CHROM agar Salmonella Plus; Graso Biotech, Poland) and incubated at 35±2°C for 24 h or at 30°C for 48–72 h. The isolation of *Salmonella* strains from tested samples was carried out in accordance with the ISO standard (PN-EN ISO 6579). Serotype identification was performed using the Rapid Identification Test for *Salmonella* Enteritidis and Typhimurium serovars (Immunolab, Poland), according to the manufacturer’s recommendations. Standard microbiological methods were used to isolate and purify bacterial strains. The strains were identified using the mass spectroscopy method (MALDI Biotyper MSP Identification Standard Methods 1.1; Bruker).

### Antimicrobial susceptibility testing

Taken together, 147 bacterial isolates were tested. Susceptibility testing was performed using the disk diffusion method (74), employing the conditions for standardization and interpretation of the results given by the Clinical and Laboratory Standard Institute (Poland). The CLSI M100 Performance Standards for Antimicrobial Susceptibility Testing, 34th Edition, were employed. The following antibiotics (Graso Biotech, Poland) were tested: penicillin (P, 10 µg per disk), ampicillin (AM, 10 µg), ampicillin-sulbactam (SAM, 10/10 µg), amoxicillin-clavulanic acid (AUG, 20/10 µg), piperacillin-tazobactam (PTZ, 100/10 µg), ticarcillin (TC, 75 µg), temocillin (TEM, 30 µg), cefoperazone (CPZ, 75 µg), ceftazidime (CAZ, 30 µg), cefotaxime (CTX, 30 µg), cefepime (CPM, 30 µg), enrofloxacin (ENF, 5 µg), ciprofloxacin (CIP, 5 µg), amikacin (AK, 30 µg), gentamicin (CN, 10 µg), streptomycin (S, 300 µg), nitrofurantoin (NI, 300 µg), colistin (CT, 50 µg), tetracycline (T, 30 µg), trimethoprim-sulfamethoxazole (TS, 1.25/23.75 µg), and clindamycin (DA, 2 µg). The choice of antibiotics was determined by the species of isolates covered by the study (a wide group of fermenting and non-fermenting bacilli and cocci). The disks were placed on a Mueller-Hinton agar (Graso Biotech, Poland), and the plates were then inverted and incubated aerobically at 37°C for 16 to 18 h. ESBL production was assessed by the method of two disks (Double-Disc Synergy Test). This test is a recommended routine practice to detect ESBL production by gram-negative bacilli. The test was performed using an amoxicillin-clavulanate (AMC, 20/10 µg) disk. The disk was placed at the center of the Mueller-Hinton agar plate, and ceftazidime (CAZ, 30 µg) and cefotaxime (CTX, 30 µg) were placed 15 mm away from the disk at the center. The plate was incubated overnight at 37°C and then examined. The presence of the keyhole phenomenon was considered a positive result for ESBL production (74).

### Detection of antibiotic-resistance genes

Rapid DNA extraction was performed using a boiling technique as described previously (75). DNA samples were either stored at 4°C (for immediate testing) or frozen at −20°C (for further procedures). PCR amplification was carried out in a final volume of 20 µL using Color Taq PCR Master Mix (EURX Ltd., Poland). The primers (Sigma-Aldrich, Germany) and PCR conditions used in this study are listed in Table S2. For β-lactam-resistance genes, PCR target genes were *bla*_CTX-M_ (76, 77), *bla*_SHV_, *bla*_TEM_ (75, 78), *bla*_CMY-2_ (6, 79) (ESBLs: extended-spectrum β-lactamases), *bla*_OXA-48_ (78), *bla*_BIC-1_, *bla*_SPM-1_, *bla*_VIM-2_, *bla*_SIM-1_, *bla*_DIM-1_, *bla*_KPC_, *bla*_NDM_, and *bla*_IPM_ (80). The non-β-lactam-resistance genes included *mcr-1*, forcolistin resistance (81), *qnrA, qnrB,* and *qnrS* for resistance to quinolone (75, 82), *aacC-2 (aac (3)-IIa*) for resistance to aminoglycosides (77, 83), and *mecA* for resistance to methicillin (84). The quality of the isolated DNA was checked using primers 27Fcm-for and 1492-rev for 16S rDNA. DNA fragments were analyzed by 1.5% agarose gel electrophoresis at 100 V for 1 h in the 0.5xTBE buffer. The Perfect Plus TM 2 kb DNA Ladder (EURX Ltd., Poland) was used as a size marker.

### Statistical analysis

For all variables, we did not find a normal distribution (in all cases, α-probabilities for the Shapiro-Wilk test were <0.001). Thus, we used a nonparametric Kruskal-Wallis test with the Dunn test (85) as *post hoc* to check for differences between numbers of four groups of bacteria, i.e., saprophytic, sporulating, thermophilic, and psychrophilic. In the case of testing differences in the number of bacteria in the cloaca, we used the Mann-Whitney test because only two groups of bacteria were identified (saprophytic and psychrophilic).

To assess whether the number of bacterial isolates in nestlings' cloaca depended on nest-specific conditions, we used a generalized linear mixed model (GLMM) with the function lmer() from the lme4 package for R. The number of cloacal bacterial isolates per nestling was used as the response variable. Nest ID was included as a fixed effect, and brood size (number of nestlings in nest) was treated as a random factor. As R_2,_ we used pseudo-R^2^Nakagava according with r.squaredGLMM() function.

For analyses of antibiotic resistance, we assigned each bacterial isolate a numeric value corresponding to the number of antibiotics to which it was resistant (from 0 to 19). Resistance profiles were compared among environmental sources (cloaca, soil, water, and food) using Kruskal–Wallis and Dunn’s *post hoc* tests. We then assessed the similarity of resistance profiles between cloacal and environmental isolates using Spearman’s rank correlation coefficients. These analyses were conducted both for individual antibiotics and for broader antibiotic classes, which we defined as follows: penicillins, penicillins with β-lactamase inhibitors, third-generation cephalosporins, fourth-generation cephalosporins, aminoglycosides, quinolones, chemotherapeutic agents, tetracyclines, and polymyxins. For all statistical tests, significance was set at *P* < 0.05. The calculations were performed in the R 4.4.4 (86), and the theoretical basis of the analyses was described previously (87).

## SUPPLEMENTAL MATERIAL

Supplemental tables spectrum.00990-25-s0001.pdf Tables S1 and S2.

ASM does not own the copyrights to Supplemental Material that may be linked to, or accessed through, an article. The authors have granted ASM a non-exclusive, world-wide license to publish the Supplemental Material files. Please contact the corresponding author directly for reuse.
