# Supplementary material for: Reassessing the role of wild birds in the spread of antibiotic resistance: the white stork as a model species in studying populations from Central European river valley
Source: Microbiol Spectr. 2025 Oct 1;13(11):e00990-25. doi: 10.1128/spectrum.00990-25 (PMC12584619; doi:10.1128/spectrum.00990-25)
Supplement: Supplemental tables — Tables S1 and S2. [file spectrum.00990-25-s0001.pdf]

SUPPLEMENTARY MATERIAL

Supplementary Table S1. pH values of the soil and water samples of feeding area of white stork.

Feeding area (abbreviation of the area name)	Soil pH	Water pH
swa	Strongly acidic soil, pH < 4.5	7.65
ch	Acidic soil, pH range: 4.6 - 5.5	7.51
gl		7.99
gr		7.64
kl		7.21
ka		7.32
d		7.67
wic		7.32
k	Slightly acidic soil, pH range: 5.6 - 6.5	7.76
m		6.95
lg		8,26
w		7.49
c		7.67
bl	Neutral soil, pH range: 6.6 - 7.2	7.53
sw		7.61
g		7.49
wl		7.08
bp	Alkaline soil, pH > 7.2	7.39
s		7.49

Abbreviations: **bl**, Brzezie Lewe; **bp**, Brzezie Prawe; **c**, Cieszów; **d**, Drągowina; **ch**, Chwalim; **g**, Gębice; **gl**, Głuchów; **gr**, Górzykowo; **k**, Krężoły; **ka**, Kargowa; **kl**, Klępina; **lg**, Leśna Góra; **m**, Mieszkowo; **s,** Sulechów; **sw**, Smolno Wielkie; **swa**, Swarzenice; **w**, Wojnowo; **wic**, Wicina; **wl**, Włostów.

Supplementary Table S2. Primers and PCR conditions used in this study.

Target	Primer	Primer sequence (5'-3')	PCR conditions	Expected amplicon size (pb)	Reference
bla$_{CTX-M}$	CTX-MU1_for	ATGTGCAGYACCAGTAARGTKATGGC	5 min at 94 °C; 30 cycles of 30 s at 94 °C, 30 s at 58°C, 1 min at 72°C; 5 min at 72°C; 4° C	593	Yousfi et al. 2018
	CTX-MU2_rev	TGGGTRAARTARGTSACCAGAAYCAGCGG			Monstein et al. 2007
bla$_{SHV}$	MultiTSO-S_for	AGCCGCTTGAGCAAATTAAAC	5 min at 94 °C; 30 cycles of 40 s at 94 °C, 60 s at 60°C, 1 min at 72°C; 5 min at 72°C; 4° C	713	Salah et al. 2019,
	MultiTSO-S_rev	ATCCCGCAGATAAATCACCAC			Dallenne et al. 2010
bla$_{TEM}$	MultiTSO-S_for	CATTTCCGTGTCGCCCTTATTC	5 min at 94 °C; 30 cycles of 40 s at 94 °C, 60 s at 60°C, 1 min at 72°C; 5 min at 72°C; 4° C	800	Salah et al. 2019,
	MultiTSO-S_rev	CGTTCATCCATAGTTGCCTGAC			Dallenne et al. 2010
bla$_{CMY-2}$	CMY-2_for	GCACTTAGCCACCTATACGGCAG	5 min at 94 °C; 30 cycles of 1 min at 94 °C, 1 min at 58°C, 1 min at 72°C; 5 min at 72°C; 4° C	758	Darwich et al. 2019
	CMY-2_rev	GCTTTTCAAGAATGCGCCAGG			Hasman et al. 2005
bla$_{OXA-48}$	MultiOXA-48_for	GCTTGATCGCCCTCGATT	5 min at 94 °C; 30 cycles of 40 s at 94 °C, 40 s at 57°C, 1 min at 72°C; 5 min at 72°C; 4° C	281	Dallenne et al. 2010
	MultiOXA-48_rev	GATTTGCTCCGTGGCCGAAA			
bla$_{BIC-1}$	BIC-F_for	TATGCAGCTCCTTTAAGGGC	5 min at 94 °C; 35 cycles of 30 s at 94 °C, 52 s at 52°C, 50 s at 72°C; 5 min at 72°C; 4° C	537	Poirel et al. 2011
	BIC-R_rev	TCATTGGCGGTGCCGTACAC			
bla$_{SPM-1}$	SPM-F_for	AAAATCTGGGTACGCAAACG	5 min at 94 °C; 35 cycles of 30 s at 94 °C, 52 s at 52°C, 50 s at 72°C; 5 min at 72°C; 4° C	271	Poirel et al. 2011
	SPM-R_rev	ACATTATCCGCTGGAACAGG			
bla$_{VIM-2}$	VIM-2_for	GATGGTGTTTGGTCGCATA	5 min at 94 °C; 35 cycles of 30 s at 94 °C, 52 s at 52°C, 50 s at 72°C; 5 min at 72°C; 4° C	390	Poirel et al. 2011
	VIM-2_rev	CGAATGCGCAGCACCAG			
bla$_{SIM-1}$	SIM-1_for	TACAAGGGATTCGGCATCG	5 min at 94 °C; 35 cycles of 30 s at 94 °C, 52 s at 52°C, 50 s at 72°C; 5 min at 72°C; 4° C	570	Poirel et al. 2011
	SIM-1_rev	TAATGGCCTGTTCCCATGTG			
bla$_{DIM-1}$	DIM-1_for	GCTTGTCTTCGCTTGCTAACG	5 min at 94 °C; 35 cycles of 30 s at 94 °C, 52 s at 52°C, 50 s at 72°C; 5 min at 72°C; 4° C	699	Poirel et al. 2011
	DIM-1_rev	CGTTCGGCTGGATTGATTTG			
bla$_{KPC}$	KPC-Fm_for	CGTCTAGTTCTGCTGTCTTG	5 min at 94 °C; 35 cycles of 30 s at 94 °C, 52 s at 52°C, 50 s at 72°C; 5 min at 72°C; 4° C	798	Poirel et al. 2011
	KPC-Rm_rev	CTTGTCATCCTTGTTAGGCG			
bla$_{NDM}$	NDM-1_for	GGTTTGGCGATCTGGTTTTC	5 min at 94 °C; 35 cycles of 30 s at 94 °C, 52 s at 52°C, 50 s at 72°C; 5 min at 72°C; 4° C	621	Poirel et al. 2011
	NDM-1_rev	CGGAATGGCTCATCACGATC			
bla$_{IPM}$	IMP-1_for	GGAATAGAGTGGCTTAAYTCTC	5 min at 94 °C; 35 cycles of 30 s at 94 °C, 52 s at 52°C, 50 s at 72°C; 5 min at 72°C; 4° C	232	Poirel et al. 2011
	IMP-1_rev	GGTTTAAYAAAACAACCACC			
mcr-1	mcr$_1$_320bp_for	AGTCCGTTTGTTCTTGTGGC	15 min at 94 °C; 30 cycles of 30 s at 94 °C, 90 s at 58°C, 1 min at 72°C; 10 min at 72°C; 4° C	320	Robelo et al. 2018
	mcr$_1$_320bp_rev	AGATCCTTGGTCTCGGCTTG			
qnrA	qnrA_for	ATTTCTCACGCCAGGATTTG	5 min at 94 °C; 30 cycles of 45 s at 94 °C, 45 s at 53°C, 1 min at 72°C; 10 min at 72°C; 4° C	516	Salah et al. 2019
	qnrA_rev	GATCGGCAAAGGTTAGGTCA			Robicsek et al. 2006
qnrB	qnrB_for	GATCGTGAAAGCCAGAAAGG	5 min at 94 °C; 30 cycles of 45 s at 94 °C, 45 s at 53°C, 1 min at 72°C; 10 min at 72°C; 4° C	469	Salah et al. 2019
	qnrB_rev	ACGATGCCTGGTAGTTGTCC			Robicsek et al. 2006
qnrS	qnrS_for	ACGACATTCGTCAACTGCAA	5 min at 94 °C; 30 cycles of 45 s at 94 °C, 45 s at 53°C, 1 min at 72°C; 10 min at 72°C; 4° C	417	Salah et al. 2019
	qnrS_rev	TAAATTGGCACCCTGTAGGC			Robicsek et al. 2006

aac(3)-IIa (*aacC2*)	fAACa_for	CGG AAG GCA ATA ACG GAG	5 min at 94 °C; 30 cycles of 30 s at 94 °C, 50 s at 50°C, 90 s at 72°C; 5 min at 72°C; 4° C	740	Yousfi et al. 2018 Gow et al. 2008
	rAACa_rev	TCG AAC AGG TAG CAC TGA G			
mecA	mecA_for	GGTGAAGTAGAAATGACTGAACGTC	5 min at 94 °C; 25 cycles of 30 s at 94 °C, 30 s at 56°C, 1 min at 72°C; 7 min at 72°C; 4° C	1160	Wang et al., 2017
	mecA_rev	GTAACGTTGTAACCACCCCAAG			
16S rRNA	27Fcm-for	AGAGTTTGATCMTGGCTCAG	3 min at 95 °C; 30 cycles of 1 min at 95 °C, 1 min at 55°C, 90 s at 72°C; 5 min at 72°C; 4° C	1500	Frank et al. 2008
	1492-rev	GGTTACCTTGTTACGACTT			

Abbreviations:

Primers: for, forward primer; rev, reverse primer

Nucleotides: Y = C or T; R = A or G; K = G or T; S = G or C; M = A or C
